# Low-level laser therapy effects on pain perception related to the use of orthodontic elastomeric separators

**DOI:** 10.1590/2176-9451.20.3.037-042.oar

**Published:** 2015

**Authors:** Rachel D'Aurea Furquim, Renata Correa Pascotto, José Rino, Jefferson Rosa Cardoso, Adilson Luiz Ramos

**Affiliations:** 1 MSc in Integrated Dentistry, Universidade Estadual de Maringá, Maringá, Paraná, Brazil; 2 Associate professor, Universidade Estadual de Maringá, Maringá, Paraná, Brazil; 3 Associate Professor, Universidade de São Paulo, São Paulo, São Paulo, Brazil; 4 Associate professor, Universidade Estadual de Londrina, Londrina, Paraná, Brazil; 5 Associate professor, Universidade Estadual de Maringá, Maringá, Paraná, Brazil

**Keywords:** Orthodontics, Laser therapy, Pain perception

## Abstract

**INTRODUCTION:**

Some patients refer to pre-banding orthodontic separation as a painful orthodontic procedure. Low-level laser therapy (LLLT) has been reported to have local analgesic effect.

**OBJECTIVE:**

The aim of this single-blind study was to investigate the perception of pain caused by orthodontic elastomeric separators with and without a single LLLT application (6J).

**METHODS:**

The sample comprised 79 individuals aged between 13 and 34 years old at orthodontic treatment onset. Elastomeric separators were placed in first maxillary molars at mesial and distal surfaces and kept in place for three days. The volunteers scored pain intensity on a visual analogue scale (VAS) after 6 and 12 hours, and after the first, second and third days. One third of patients received laser applications, whereas another third received placebo applications and the remaining ones were controls. Applications were performed in a split-mouth design. Thus, three groups (laser, placebo and control) were assessed.

**RESULTS:**

No differences were found among groups considering pain perception in all periods observed.

**CONCLUSION:**

The use of a single-dose of LLLT did not cause significant reduction in orthodontic pain perception. Overall pain perception due to orthodontic separator placement varied widely and was usually mild.

## Abstract (pt)

**INTRODUÇÃO:**

alguns pacientes referem-se à separação ortodôntica pré-bandagem como um procedimento doloroso. Tem sido relatado que a terapia com *laser*de baixa intensidade (LLLT) produz um efeito analgésico local.

**OBJETIVO:**

o objetivo deste estudo simples-cego foi investigar a percepção da dor causada por elásticos ortodônticos separadores, com ou sem uma única aplicação de LLLT (6J).

**MÉTODOS:**

a amostra foi composta por 79 indivíduos com 13-34 anos de idade no início do tratamento ortodôntico. Elásticos separadores foram colocados nos molares superiores, nas proximais mesial e distal, e mantidos por três dias. Os voluntários marcaram a intensidade da dor em uma escala visual analógica (EVA) após 6 horas, 12 horas, 1 dia, 2 dias e 3 dias. Um terço dos dentes separados recebeu aplicações de *laser*; outro terço, aplicações placebo; e os demais foram usados como controle. As aplicações foram realizadas segundo um desenho metodológico de boca dividida. Portanto, foram comparados três grupos: *laser*, placebo e controle.

**RESULTADOS:**

não foram encontradas diferenças entre os grupos, em relação à percepção de dor, em nenhum dos períodos observados.

**CONCLUSÕES:**

a utilização da LLLT em dose única não causou redução significativa na dor ortodôntica. Além disso, a percepção geral da dor devida à colocação de separadores ortodônticos variou muito e foi, geralmente, leve.

## INTRODUCTION

Pain is often associated with dental procedures. It has been reported that 28% of orthodontic patients consider discontinuing treatment due to fear of pain, while 39% of them claim it is the worst feature of orthodontic appliances.^1^ After placement of orthodontic accessories, such as elastomeric separators, archwires or activation loops, the affected areas undergo a painful process triggered by pressure and stress.^2^^,^ 3 Although pain is subjective and may vary among individuals, studies show that all patients, regardless of age, have reported some degree of pain during treatment.^2^^,^ 3

It has been observed that, due to being mild to moderate and often transient pain,^4^ medications are not routinely prescribed in orthodontic practice, unless discomfort becomes intolerable.^5 ^Moreover, medications can produce side effects and are contraindicated for allergic patients.^6^^,^ 7 Low-level laser therapy (LLLT) has been reported to reduce inflammation and pain by reducing prostaglandin and interleucine production;^7^ and has, therefore, been proposed as an alternative analgesic in Dentistry.^6^^-^ 14 However, few clinical LLLT trials^15^ have been performed with clear methods, significant samples, homogeneous groups and a placebo group. Furthermore, it is not clear to what extent the use of pre-banding elastomeric orthodontic separators is perceived by patients as painful.

In light of the above, the aim of this study was to assess pain perception associated with elastomeric separators with and without a single application of 808-nm LLLT.

## MATERIAL AND METHODS

This study was approved by Universidade Estadual de Maringá Institutional Review Board (0315.0.093.000-09) and all volunteers and legal guardians signed an informed consent form.

Sample size calculation was performed with a confidence level of 95%, 5-mm margin of error, 8.1 mm standard deviation, and an infinite population.^9^ Although the results showed that each group should comprise 11 individuals, 25 subjects were initially assigned to each group, given the inclusion of the placebo group and the clinical nature of the research.

The following inclusion criteria were applied: complete permanent dentition in the maxillary arch, except for third molars, and good systemic health. Patients who had undergone prior oral LLLT; those who presented with systemic problems, such as diabetes or metabolic diseases, which may interfere in the inflammatory process; pregnant or lactating patients; those who were using painkillers or anti-inflammatory medications and/or presented with clear signs of periodontal disease, such as bleeding or signs of inflammation (pain, heat, swelling and redness) were excluded from the study.

The initial sample comprised 100 patients and all of them had the following maxillary teeth separated with elastomeric separators (Morelli - Sorocaba, SP, Brazil): between the second premolar and first molar (mesial of first molar), and between the first molar and second molar (distal of first molar).^6^^,^ 12

Patients were randomly divided into four initial groups in which maxillary molars on both sides received elastomeric separators. Each group was approached differently, as follows: Group 1, LLLT applied on the left side and placebo on the right side (blind) (SOLce); Group 2, LLLT applied on the left side and control on the right side (aware) (SOLci); Group 3, control on the right side and placebo on the left side (blind) (SOce); Group 4, control on both sides (aware) (SOci). The term "blind" refers to the fact that patients were not aware of the procedure (placebo).

In the group "orthodontic separation with laser application (blind)" (SOLce), LLLT was applied immediately after elastomeric separators placement in the maxillary left first molars. On the right side, placebo applications were performed, with the LLLT device producing beeps without firing the laser. Since the infrared laser used is not visible and protection glasses were on, patients could not detect any differences between the two applications.

In the group "orthodontic separation with laser application (aware)" (SOLci), laser therapy was performed only on the left side, as in group 1; but this time, patients were aware that the laser would be applied on one side, only. On the other side, no placebo applications were performed.

In the group "orthodontic separation (blind)" (SOce), recorded as group 3, no LLLT was applied. However, on the left side, placebo applications were performed as previously described. Patients did not receive laser applications on the other side. Thus, the psychological factor was assessed in terms of what extent to which it interferes in the pain process, inducing the patient into thinking that the side supposedly treated with some sort of therapy would hurt less.

In the group "orthodontic separation (aware)" (SOci), recorded as group 4, the volunteers received neither placebo nor laser applications, thus fully characterizing it as the control group.

Twenty-one subjects dropped out of the study or provided incorrect data: five of them reported severe pain (two from the SOLce group, one from the SOce group and two from the SOci group); and sixteen lacked complete data in one of the study periods (three from the SOce group and 13 from the SOci group). Therefore, final data distribution (n = 79) was as follows: SOLce (n = 23), SOLci (n = 25), SOce (n = 21) and SOci (n = 10).

Considering the sample in terms of the sides assessed (n = 158), distribution was as follows: laser = 30.37% (n = 48), placebo = 27.48% (n = 44), control = 41.77% (n = 66).

Applications were performed with a Whitening Lase II device (DMC Equipment Ltda., São Carlos, Brazil) which has two laser probes with distinct functions: a smaller laser probe for LLLT and a curved laser probe for teeth bleaching. The laser therapy probe in infrared mode (AsGaAl) was used.

**Figure 1.**
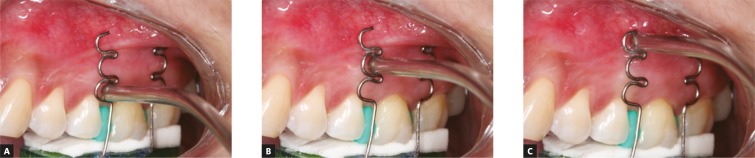
Guide and scheme of laser applications used in the study. A) 10-second application in the mesio-cervical region; B) 10-second application in the mesio-medial region; C) 10-second application in the mesio-apical region. The three regions (cervical, medial and apical) also received laser applications distally, thereby totaling 60 seconds per tooth (6 J / tooth).

A standard guide was used for all patients (after disinfection with 70% alcohol and protection with film paper in the foam area) based on the average size (13 mm) of the buccal roots of the maxillary first molar.^16^ The device was placed on the occlusal surface of teeth and supported between the marginal ridges of the teeth involved. The guide was fabricated so that the first application was performed 5 mm above the gingival papilla, approaching patient's bone crest region. The total length of the guide was 12 mm, allowing three applications, 4 mm apart from each other, to be performed (Fig 1).

The wavelength used was 808 nm, with a fluency of 80 J/cm^2^, as recommended by the manufacturer (DMC Equipment Ltda., São Carlos, Brazil), thereby totaling approximately 6 J of energy per tooth (1 x 60 s x 100 mW).The probe of the device remained in contact with the gingival tissue during applications. Elastomeric separators were placed and laser applications performed by the same previously trained and calibrated operator.

Subsequently, all patients were instructed to rate their level of spontaneous pain on a visual analogue scale (VAS). Initial scores were assigned as soon as the patient arrived at the office and before any procedure was carried out. This initial score made it possible to judge whether or not the patient already felt some pain, which was not related to the separation procedure, in the teeth involved in the study. After separation, patients' pain levels were recorded 6 hours, 12 hours and 1, 2 and 3 days following separation. The scores assigned by the patient on the visual analogue scales were measured with a caliper (Mitutoyo, Japan). A zero score, located on the left side of the scale, suggested no pain; while a 100 (100 mm) score, at the right end of the scale, suggested maximum pain. The center of the scale corresponded to a score equal to 50 and suggested moderate pain. This information was provided to the subjects before they started assigning scores on their dental history cards, which patients took home.

Data were tested for normality of distribution by means of the Shapiro-Wilk test. Should normal distribution not be found, data were presented using median and their quartiles (1^st^ and 3^rd)^. Pain perception was assessed by analysis of variance (ANOVA) for repeated measures. Mauchly's sphericity test was also applied and, whenever violated, technical corrections were performed by Greenhouse-Geisser test. Statistical significance was set at 5% and analyses were carried out by means of SPSS version 15.0.

## RESULTS

Patients' mean age was 23.4 ± 6.3 years for group SOLce (9 men and 14 women); 22.3 ± 4.1 years for group SOLci (8 men and 17 women); 23 ± 4.7 years for group SOce (6 men and 15 women) and 25.5 ± 7.8 years for group SOci (1 man and 9 women) (Table 1).

**Table 1.** Demographic analysis of group data.

	SOLce	SOLci	SOce	SOci
	(n = 23)	(n = 25)	(n = 21)	(n = 10)
Age (years) (mean ± SD)	23.4 ± 6.3	22.3 ± 4.1	23 ± 4.7	25.5 ± 7.8
Sex				
Male - n (%)	9 (39.1%)	8 (32%)	6 (28.6%)	1 (10%)
Female - n (%)	14 (60.9%)	17 (68%)	15 (71.4%)	9 (90%)*

* p < 0.05.

Data frequency distribution for age and sex was performed in a similar manner (p > 0.05), confirming the homogeneity of the sample. Female patients were predominant only in the control group (Table 1). This fact did not hinder comparison among the laser, placebo and control sides (Tables 2 and 3).

**Table 2.** Median and median quartiles (1st - 3rd) of the SOLce, SOLci, SOce, SOci groups in all periods analyzed, comparing left and right sides.

	SOLce		SOLci		SOce		SOci	
	(n = 23)		(n = 25)		(n = 21)		(n = 10)	
	Left side (laser)	Right side (placebo light)	Left side (laser)	Right side (no light)	Left side (placebo light)	Right side (no light)	Left side (no light)	Right side (no light)
	Md (1^st ^– 3^rd^)	Md (1^st ^– 3^rd^)	Md (1^st ^– 3^rd^)	Md (1^st ^– 3^rd^)	Md (1^st ^– 3^rd^)	Md (1^st ^– 3^rd^)	Md (1^st ^– 3^rd^)	Md (1^st ^– 3^rd^)
6 h	1.2 (0 – 12.4)	0.9 (0 – 11.8)	0 (0 – 8)	2.7 (0 – 21.8)	1.4 (0 – 19.9)	3.1 (0 – 12.6)	3.6 (0 – 12.9)	1.7 (0 – 12.2)
12 h	4.5 (0 – 23.3)	2.5 (0 – 16)	3 (0 – 10.8)	4.2 (0 – 11.2)	0.49 (0 – 22.7)	1.3 (0 – 9.3)	4.5 (0.8 – 7)	4.1 (0 – 7.2)
1 day	4.8 (0 – 18.3)	2.4 (0 – 16.1)	2.4 (0 – 23.6)	3.2 (0 – 26.7)	1.3 (0 – 24.8)	0.9 (0 – 19.5)	1.6 (0 – 4.8)	1.8 (0.5 – 6.5)
2 days	3.2 (0 – 11.8)	0 (0 – 12.5)	4.5 (0 – 10.7)	4 (0 – 17.8)	0 (0 – 11.7)	0.8 (0 – 12.8)	1.4 (0 – 6.2)	1.9 (1.1 – 4.3)
3 days	0 (0 – 6.3)	0 (0 – 3.3)	0.5 (0 – 9.1)	0.8 (0 – 13.7)	0 (0 – 8.4)	0 (0 – 5.8)	0 (0 – 5.4)	0.5 (0 – 3.1)

Md = median; (1st - 3rd) = first and third quartiles; F Greenhouse-Geisser test= 1.78; p = 0.16.

**Table 3.** Median and median quartiles (1st - 3rd) of scores side by side with laser, placebo and control sides applications in all periods analyzed.

	Laser (n = 44)	Placebo (n = 44)	Control (n = 66)
	Md (1^st^ – 3^rd^)	Md (1^st^ – 3^rd^)	Md (1^st^ – 3^rd^)
6 h	0.6 (0 – 8.3)	1.1 (0 – 8)	2.9 (0 – 14.8)
12 h	4.2 (0 – 13.6)	1.7 (0 – 17.7)	3.4 (0 – 10.7)
1 day	2.3 (0 – 18.6)	1.9 (0 – 22.3)	1.7 (0 – 19.8)
2 days	2.8 (0 – 11)	0 (0 – 11.6)	2.9 (0 – 12.9)
3 days	0 (0 – 6.4)	0 (0 – 6.5)	0.1 (0 – 6.2)

Md = median; (1st - 3rd) = first and third quartiles; F Greenhouse-Geisser test = 1.16; p = 0.32.

All volunteers assigned zero to pain perception score at baseline. Among the 79 volunteers, 12.65% (n = 10) did not report any pain over all evaluated periods; and only 15.18% (n = 12) reported pain levels equal to or greater than 40 in at least one of the assessment periods. No statistical difference was found (*p *= 0.16) between left and right sides in all periods compared across all groups (Table 2). Although the median was low, the pain peak perceived by patients occurred between 12 hours and 1 day (Tables 2 and 3).

LLLT applications, placebo applications and control sides were compared during the scoring periods. The three situations showed no statistical difference (*p *= 0.32) in terms of pain level (Table 3).

## DISCUSSION

Corroborating the results of previous studies,^2^^,^ 3 the pain caused by orthodontic procedures (separators or leveling archwires) reaches its peak 12 and 24 hours after placement (Table 3). However, in this study, pain perception, as shown in VAS scores, was highly variable, with a relatively low median. It is a known fact that separators cause pain. Despite reports by some people who do not feel any pain whatsoever,^6^ most authors report that, although pain intensity or location may vary, all patients eventually complain, which indicates that the procedures performed in orthodontic practice are always a nuisance.^2^^,^ 3 ^,^ 4 ^,^ 7 In the present study, 12.65% (n = 10) did not report any pain and only 15.18% (n = 12) reported pain levels equal to or greater than 40. If the five volunteers who dropped out of the study after reporting too much pain were to be included, this percentage would rise to 18% of the initial sample. Those distributions related to pain were similar among groups. Therefore, patients who claimed that the pain caused by orthodontic separation was relevant represented a minority of the sample. It is worth noting that the effects of LLLT could only be noted if the majority of subjects had perceived increased pain. Nevertheless, a detailed assessment of patients reporting pain greater than or equal to 40 on VAS, in at least one of the periods, revealed that six of them reported feeling greater pain on the laser side, compared to placebo or control, while six of them assigned lower scores to the laser side.

Although pain is seen as a subjective and, therefore, hard-to-assess variable, the use of visual analogue scales, as it was the case in this study, has been widely reviewed and is nowadays regarded as a reliable method.^6^^,^ 9 ^,^ 17 In comparison to other investigations on orthodontic pain perception, the present study disclosed lower VAS score values. Fujiyama et al^12^ reported higher scores that reached 80, 12 and 24 hours after placing separators and when no laser was applied; and 40 when it was applied; however, no placebo group was used. Our study corroborates that pain registered in VAS scores varies from mild to moderate.^18^^-^ 23

It is worth noting that, as performed in a variety of other studies,^6^^,^ 7 ^,^ 11 ^,^ 12 ^,^ 18 volunteers were asked to score spontaneous pain; however, other authors registered other situations, such as biting, to which patients sometimes referred as being more painful than a spontaneous symptom.^22^^,^ 24

In the present study, a split-mouth, single-blind model was adopted and a placebo side was included, which allowed the authors to compare intrasubject pain perception with and without LLLT. Lim et al^6^ conducted a similar study with separators and found no difference between the placebo and laser sides. Additionally, their scores were similar to those found in the present study, which also shows considerable variability.^6 ^Those data also corroborate a recent study performed by Abtahi et al.^18^

Youssef et al,^13^ Tortamano et al,^14^ Turhani et al^11^ and Harazaki et al,^7^ for instance, applied laser in patients undergoing orthodontic treatment. The authors assessed pain during alignment and leveling or when performing canine retraction. Given that these procedures involve a higher number of teeth, they may enhance pain perception and underscore LLLT effects. Thus, it does not seem reasonable to compare these results with the present study which assessed pain perception in the presence of elastomeric separators.

A wide range of laser types, with different wavelengths and energy doses, can be found in the literature. AsGaAl diode laser, used in studies by Youssef et al,^13^ Tortamano et al^14^ and Lim et al,^6^ was also used in the present study. Moreover, Harazaki et al^7 ^used HeNe laser whereas Fujiyama et al^12^ used CO_2_ laser. At lower wavelengths, for instance, 632.8 nm^7^ and 670 nm^11^, no difference, in terms of pain intensity, was reported between groups with or without laser applications. Nevertheless, the use of high-level laser, with wavelength of 808 nm, revealed statistically significant pain reduction in some studies.^13^^,^ 23 This was the wavelength used in the present study, following the manufacturer's recommendations. However, even the use of laser with wavelength at 830 nm has yielded discrepant results, with LLLT producing some analgesic effect,^14 ^despite not being significant.^6^

According to the manufacturer's instructions, we used, in this study, 6 J of energy in a single dose. Other similar studies used from 5 to 12 J of energy in single or daily applications. One single application seems more practical, as it does not rely on further appointments and patient cooperation.^19^. Although the amount of energy probably influences the analgesic effect, some studies report LLLT efficacy^19^^-^ 22 or not^6^^,^ 18 with similar energy and frequency levels. Further studies can clarify this point.

A systematic review has recently reported that nonsteroidal anti-inflammatory drugs (NSAIDs), such as COX-2 selective inhibitor, are still the best choice to reduce pain during orthodontic treatment, despite potential side effects.^15^ Another recent study revealed that a single dose of Piroxicam, taken 60 minutes before separator placement, reduces pain.^24^

Since patients generally perceive pain as mild and transient, an analgesic regimen should only be adopted for less tolerant patients. However, should such regimen prove necessary, a single application of LLLT does not seem to provide a fully effective protocol for this purpose.

## CONCLUSION

A single application (6 J) of LLLT (808 nm) did not produce significant effects on the perception of pain caused by orthodontic separation.

Overall, pain arising from the use of orthodontic pre-banding elastomeric separators was low and transient, and discomfort was reported as relevant only by a minority of patients (18% in this study).
